# Burnout and psychological distress amongst Australian healthcare workers during the COVID-19 pandemic

**DOI:** 10.1177/1039856220965045

**Published:** 2020-10-12

**Authors:** Hannah Dobson, Charles B Malpas, Aidan JC Burrell, Caroline Gurvich, Leo Chen, Jayashri Kulkarni, Toby Winton-Brown

**Affiliations:** Department of Psychiatry, The Alfred Hospital, Melbourne, VIC, Australia; The Monash Alfred Psychiatry Research Centre, Monash University, VIC, Australia; Department of Neuroscience, Central Clinical School, Monash University, Melbourne, VIC, Australia; Department of Intensive Care, The Alfred Hospital, Melbourne, VIC, Australia; The Monash Alfred Psychiatry Research Centre, Monash University, Melbourne, VIC, Australia; The Monash Alfred Psychiatry Research Centre, Monash University, Melbourne, VIC, Australia; The Monash Alfred Psychiatry Research Centre, Monash University, Melbourne, VIC, Australia; Department of Psychiatry, The Alfred Hospital, Melbourne, VIC, Australia; Department of Neuroscience, Central Clinical School, Monash University, VIC, Australia

**Keywords:** healthcare workers, mental health, burnout, COVID-19

## Abstract

**Objective:**

To examine psychological distress in healthcare workers (HCWs) during the COVID-19 pandemic in April–May 2020.

**Methods:**

A cross-sectional survey examining demographic, employment and mental health characteristics of HCWs in a large metropolitan hospital in Australia.

**Results:**

HCWs showed significant symptoms of moderate-severe level depression (21%), anxiety (20%) and posttraumatic stress disorder (PTSD; 29%), associated with burnout, prior psychiatric history, profession and resilience.

**Conclusion:**

Despite low levels of COVID contact, moderate to high levels of psychological distress were reported. Continued monitoring and support for HCWs’ mental well-being is warranted as the COVID-19 pandemic develops.

In December 2019, Wuhan reported a novel pneumonia, known as coronavirus disease 2019 (COVID-19). As of 20 July 2020, over 14 million were confirmed cases worldwide, with over 500,000 fatalities.^1^ In response to this global pandemic, Australia rapidly implemented strict social distancing policies, and hospitals were modified to respond to a potential surge in COVID-19 presentations. As of 20 July 2020, Australia has had 11,802 cases and 122 fatalities.^1^

Healthcare workers (HCWs) exhibit higher rates of anxiety, depression and suicidal ideation when compared to the general population.^2,3^ Burnout, a syndrome of exhaustion, detachment and reduced fulfilment, develops in 20%–80%^4^ of HCWs. During emergencies such as pandemics, increased posttraumatic stress disorder (PTSD), anxiety and depression in HCW^5^ may relate to both individual and system factors.^6^

Recent data have identified high levels of anxiety, depression, and insomnia amongst HCW during the COVID-19 pandemic.^7–9^ However, little is known about the psychological impact of the COVID-19 pandemic on Australian HCWs or comparable countries where healthcare resources are not overwhelmed.

This study measured self-reported symptoms of anxiety, depression and PTSD in HCWs at a tertiary centre undergoing workforce restructuring and mandated social distancing in Australia. We explored relationships with predictive and mediating factors including COVID-19 exposure, profession, past psychiatric history and measures of burnout.

## Methods

A cross-sectional survey was conducted between 16 April and 13 May 2020 amongst staff at a major tertiary hospital in Melbourne, Australia. In anticipation of the COVID-19 pandemic, major hospital preparations including ward repurposing and staff redeployment occurred alongside mandated restriction of social contact outside hospital. The survey closed at a time that initial social distancing practices were being relaxed in Australia.

The Hospital Ethics Committee approved the study (project ID 204/20). Participants were recruited via well-being workshops in front-line departments, followed by targeted emails, posters and word of mouth. Written information was provided to participants and electronic consent obtained. The survey was anonymous; however, participants had the opportunity to engage with a psychiatric clinician following the survey.

Demographic data were reported, including occupation (senior medial staff, junior medical staff, nursing, allied health, other), gender (male, female, non-binary), age, past psychiatric history and years of experience. Participants who reported direct contact with COVID-19 patients were defined as ‘front-line’. ‘High-exposure environments’ were classified as the emergency department (ED), intensive care unit (ICU), respiratory medicine and infectious diseases departments.

The primary outcome was self-reported levels of psychological distress (symptoms of depression, anxiety and PTSD) experienced during the 2 weeks prior to the survey. The nine-item Patient Health Questionnaire (PHQ-9; range 0–27),^10^ seven-item Generalised Anxiety Disorder scale (GAD-7; range 0–21),^11^ 22-item Impact of Event Scale–Revised (IES-R; range 0–88),^12^ 10-item Connor-Davidson Resilience Scale (CD-RISC10, range 0–40),^13^ and Stanford Professional Fulfilment Index (PFI; burnout range 0–40)^14^ were used to assess the severity of symptoms of depression, anxiety, PTSD, resilience and burnout, respectively. The total scores of these instruments were interpreted as per previously validated cut-offs.^10–14^

We calculated descriptive statistics, including means and percentages. Contingency table analyses, using χ^2^ tests of independence, were used to investigate the relationship between nominal variables, and *t*-tests were used for continuous variables. General linear models (GLMs) were used to investigate the predictors of psychological outcome variables. Continuous variables were centred prior to analysis and categorical variables were dummy-coded after choosing a sensible reference class. Omnibus *F* tests were used for null hypothesis significance testing of overall model effects, with effect sizes reported as partial eta squared (η^2^_p_) Unstandardised model coefficients with 95% confidence intervals were computed for key models. Care was taken to assess for statistical assumptions, including the distribution of model residuals where necessary. All statistical analyses were performed using Jamovi (version 1.2)^15^ and R (version 3.6.3).^16^

## Results

During the study period, 406 responses were recorded; 86 were excluded as no psychometric scales were completed. The final sample included 320 participants. This included 99 medical practitioners (31%, 58 senior medical staff, 41 junior medical staff), 84 nurses (26%), 105 allied health practitioners (33%) and 28 non-clinical or other (9%). The majority of participants were female (78%).

Two hundred and forty-one participants (75%) had at least 5 years of clinical experience and 121 participants (39%) were front-line workers. Medical and nursing staff were more likely to be in direct contact with COVID-19 patients than other professions (χ^2^(4) = 81.7, *p* < 0.001, Table 1).

**Table 1.** Sample characteristics

	No. (%)					
	Total	Senior medial staff	Junior medical staff	Nursing	Allied health	Non-clinical/Other
**Overall**	320 (100)	58 (18)	41 (13)	86 (27)	105 (33)	26 (8)
**Gender**						
Male	58 (18.4)	23 (39.7)	19 (46.3)	8 (9.3)	7 (6.7)	1 (3.8)
Female	248 (78.5)	34 (58.6)	22 (53.7)	74 (86)	93 (88.6)	25 (96.2)
Not-specified/Other	10 (3.2)	1 (1.7)	0 (0)	4 (4.7)	5 (4.8)	0 (0)
**Age**						
19–29	75 (23.7)	0 (0)	22 (53.7)	21 (24.4)	26 (24.8)	6 (23.1)
30–39	100 (31.6)	16 (27.6)	17 (41.5)	25 (29.1)	36 (34.3)	6 (23.1)
40–49	69 (21.8)	17 (29.3)	2 (4.9)	22 (25.6)	23 (21.9)	5 (19.2)
50 or over	72 (22.8)	25 (43.1)	0 (0)	18 (20.9)	20 (19)	0 (34.6)
**Years of experience**						
0–5 years	77 (24.4)	3 (5.2)	26 (63.4)	12 (14)	25 (24)	11 (42.3)
6–10 years	91 (28.9)	18 (31)	13 (31.7)	33 (38.4)	23 (22.1)	4 (15.4)
11–15 years	50 (15.9)	9 (15.5)	2 (4.9)	12 (14)	19 (18.3)	8 (30.8)
>15 years	97 (30.8)	28 (48.3)	0 (0)	29 (33.7)	37 (35.6)	3 (11.5)
**Front-line**	121 (38.7)	30 (52.6)	25 (61)	54 (62.8)	11 (10.7)	1 (3.8)
**High exposure environment**	128 (52)	26 (20.3)	20 (52.6)	63 (74.1)	12 (11.5)	7 (26.9)
**Past psychiatric diagnosis**	97 (30.7)	10 (17.2)	13 (31.7)	34 (39.5)	27 (25.7)	13 (50)

Ninety-eight participants (31%) reported a previous psychiatric diagnosis. Senior medical staff were less likely to report a prior psychiatric diagnosis compared with other professions (χ^2^(4) = 13.9, *p* = 0.008, Table 1).

A subset of participants screened positively for moderate-to-severe symptoms of depression (21%), anxiety (20%), and PTSD (29%; Table 2). Twenty-three participants (8.1%) reported suicidal ideation during the 2-week reporting period; 18 participants (6.3%) requested follow-up by a psychiatric clinician.

**Table 2.** Severity categories of depression, anxiety and posttraumatic stress in total cohort and subgroups

	No. (%)										
	Occupation					Gender			Working position		
Overall	Senior medical staff	Junior medical staff	Nursing	Allied health	Other	Male	Female	Other/Not specified	Front-line	Second-line	
**GAD-7, anxiety**											
Minimal or none	136 (48.1)	29 (54.7)	17 (50)	33 (42.9)	51 (53.1)	6 (26.1)	30 (61.2)	104 (45.6)	3 (33.3)	51 (47.2)	84 (48.3)
Mild	89 (31.4)	17 (32.1)	10 (29.4)	25 (32.5)	27 (28.1)	10 (43.5)	13 (26.5)	74 (32.5)	3 (33.3)	39 (36.1)	51 (29.3)
Moderate	42 (14.8)	7 (13)	5 (14.7)	13 (16.9)	13 (13.5)	4 (17.4)	4 (8.2)	37 (16.2)	2 (22.2)	16 (14.8)	25 (14.4)
Severe	16 (5.7)	0 (0)	2 (5.9)	6 (7.8)	5 (5.2)	3 (13)	2 (4.1)	13 (5.7)	1 (11.1)	2 (1.9)	14 (8)
**PHQ-9, depression**											
Minimal or none	142 (50.4)	31 (58.5)	19 (55.9)	36 (46.8)	49 (51.6)	7 (30.4)	26 (53.1)	111 (48.9)	6 (66.7)	57 (52.8)	85 (49.1)
Mild	82 (29.1)	18 (34)	8 (23.5)	21 (27.3)	27 (28.4)	8 (34.8)	13 (26.5)	68 (30)	2 (22.2)	32 (29.6)	49 (28.3)
Moderate	35 (12.4)	3 (5.7	5 (14.7)	12 (15.6)	13 (13.7%)	2 (8.7)	4 (8.2)	31 (13.7)	0 (0)	12 (11.1)	22 (12.7)
Severe	23 (8.2)	1 (1.9)	2 (5.9)	8 (10.4)	6 (6.3)	6 (26.1)	6 (12.2)	17 (7.5)	1 (11.1)	7 (6.5)	17 (9.8)
*Reported suicidal ideation*	22 (7.8)	3 (5.7)	5 (14.7)	7 (9.2)	2 (2.1)	5 (21.7)	15 (6.6)	9 (16.3)	0 (0)	7 (6.5)	15 (8.7)
**IES-R, posttraumatic stress disorder**											
Minimal or none	57 (19.8%)	14 (26.4)	7 (20)	12 (15.6)	20 (20.2)	4 (16.4)	14 (27.5)	42 (18.2)	1 (11.1)	21 (19.1)	36 (20.3)
Mild	147 (51)	32 (60.4)	12 (34.3)	45 (58.4)	50 (50.5)	8 (33.3)	28 (54.9)	116 (50.2)	4 (44.4)	61 (55.5)	84 (47.5)
Moderate	79 (27.4)	7 (13.2)	15 (42.9)	18 (23.4)	28 (28.3)	11 (45.8)	7 (13.7)	71 (30.7)	3 (33.3)	27 (24.5)	53 (29.9)
Severe	5 (1.7)	0 (0)	1 (2.9)	2 (2.6)	1 (1)	1 (4.2)	2 (3.9)	2 (0.9)	1 (11.1)	1 (0.9)	4 (2.3)

*Note.* GAD-7 = seven-item Generalised Anxiety Disorder; IES-R = Impact of Event Scale–Revised; PHQ-9 = nine-item Patient Health Questionnaire.

Eighty-three participants (29.5%) screened positively for symptoms of burnout. Rates of burnout, depression, anxiety and PTSD differed across the professions sampled; senior medical staff reported the lowest levels of psychological distress.

Front-line workers reported high levels of resilience when compared with other HCWs and no greater severity of psychological distress (Table 3). Working in a high-exposure environment was associated with greater endorsement of symptoms of PTSD (*t*(279) = 2.26), *p* = 0.024) and burnout (*t*(270) = 2.03, *p* = 0.044).

**Table 3.** Measures of psychological distress by profession and working position

	Profession (mean, SD)								Working position (mean, SD)				
	Senior medical staff	Junior medical staff	Nursing	Allied health	Other	*F*	*p*	η^2^_p_	Front-line	Second-line	*F*	*p*	η^2^_p_
Burnout	0.63 (0.52)	1.03 (0.79)	1.13 (0.78)	1.02 (0.79)	1.08 (0.89)	3.97	0.004	0.055	0.95 (0.75)	1.01 (7.78)	0.51	0.48	0.002
Depression	4.09 (3.57)	5.59 (5.25)	6.61 (6.10)	5.56 (4.51)	9.78 (6.87)	5.40	<0.001	0.072	5.48 (5.09)	6.23 (5.52)	1.3	0.26	0.005
Anxiety	4.53 (3.71)	5.74 (4.96)	6.57 (5.08)	5.51 (4.49)	7.87 (5.79)	2.70	0.031	0.037	5.53 (4.25)	6.02 (5.03	0.71	0.40	0.004
PTSD	14.5 (9.48)	22.3 (11.6)	20.5 (11.2)	18.9 (11.4)	24.7 (13.2)	4.65	0.001	0.062	18.7 (10.4)	19.9 (12.2)	0.74	0.39	0.003
Resilience	29.9 (5.15)	27.9 (4.86)	29.0 (5.68)	27.5 (5.56)	28.1 (6.02)	1.99	0.096	0.026	29.8 (5.2)	27.7 (5.61)	10.8	0.001	0.035

*Note.* PTSD = posttraumatic stress disorder.

Burnout was associated with greater endorsement of anxiety (*F*(1,254) = 71.63 *p* < 0.001, η^2^_p_ = 0.22), depression (*F*(1,254) = 74.56 *p* < 0.001, η^2^_p_ = 0.23) and PTSD symptoms (*F*(1,251) = 56.92 *p* < 0.001, η^2^_p_ = 0.19). Resilience was associated with less endorsement of anxiety (*F*(1,254) = 17.48 *p* < 0.001, η^2^_p_ = 0.06), depression (*F*(1,254) = 19.00 *p* < 0.001, η^2^_p_ = 0.07) and PTSD symptoms (*F*(1,251) = 10.26 *p* = 0.002, η^2^_p_ = 0.04). A pre-existing psychiatric history was associated with a greater endorsement of depression symptoms and burnout (*F*(1,254) = 6.22 *p* = 0.13, η^2^_p_ = 0.02; *F*(1,255) = 7.89 *p* = 0.005, η^2^_p_ = 0.03; Table 4).

**Table 4.** Relationship between resilience, past psychiatric history, burnout and psychological distress

	GAD-7, anxiety	PHQ-9, depression	IES-R, posttraumatic stress disorder	PFI, burnout
Resilience	**−0.19[−0.29, −0.10]**	**−0.23 [−0.33, −0.13]**	**−0.37 [−0.60, −0.14]**	**−0.04 [−0.06, −0.03]**
Age	−0.13 [−0.47, 0.22]	−0.03 [−0.40, 0.35]	0.57 (−0.30, 1.43]	−0.03 [−0.10, 0.03]
Profession allied health – other	**−2.13 [–3.95, –0.32]**	−3.89 [–5.87, –1.90]	−4.85 [–9.41, –0.29]	−0.03 [–0.36, 0.30]
Junior medical staff – Other	−1.79 [–4.01, 0.43]	**−3.63 [–6.06, –1.21]**	−1.55 [–7.11, 4.02]	−0.17 [–0.58, 0.24]
Nursing – Other	−1.06 [–2.98, 0.86]	**–3.04 [–5.14, –0.94]**	−3.31 [–8.13, 1.52]	0.16 [–0.20, 0.51]
Senior medical staff – Other	−1.04 [–3.09, 1.01]	**–3.36 [–5.59, –1.12]**	−5.12 [–10.30, 0.06]	−0.22 [–0.60, 0.15]
Years of experience	0.17 [–0.48, 0.82]	0.24 [–0.47, 0.95]	−0.60 [–2.23, 1.03]	−0.07 [–0.19, 0.05]
Front-line	−0.15 [–1.44, 1.16]	−0.11 [–1.53, 1.31]	0.74 [–2.54, 4.01]	0.14 [–0.10, 0.38]
Working in a high exposure environment	−0.21 [–1.48, 1.06]	0.13 [–1.26, 1.52]	−1.54 [–4.72, 1.65]	**–0.23 [–0.46, –0.00]**
Past psychiatric history	1.05 [–0.01, 2.11]	**1.46 [0.31, 2.62]**	2.14 [–0.53, 4.81]	**0.27 [0.08, 0.46]**
Burnout	**2.89 [2.22, 3.56]**	**3.22 [2.49, 3.96]**	**6.46 [4.77, 8.14]**	

*Note*. Unstandardised model coefficients [95% confidence intervals] from separate general linear models (GLMs). Bold values indicate those where intervals did not capture 0.

GAD-7 = seven-item Generalised Anxiety Disorder; IES-R = Impact of Event Scale–Revised; PHQ-9 = nine-item Patient Health Questionnaire; PFI = Stanford Professional Fulfilment Index.

## Discussion

We report the first study, to our knowledge, of mental health outcomes amongst Australian HCWs during the COVID-19 pandemic. A substantial proportion of HCWs self-reported moderate-to-severe symptoms of depression, anxiety and PTSD (21%, 20% and 29%, respectively). This was comparable to published rates reported by countries severely affected by the COVID-19 pandemic,^8,9^ and to those reported in the Australian public around the same time.^17^

Similar to these reports, this study doesn’t allow comparison to pre-pandemic baseline data. However, previous research suggests HCWs experience higher rates of anxiety and depression when compared with the general population.^2,3^

Psychological distress in HCW may develop in response to a range of stressors: risk of personal infection, fear of spreading the illness to family and friends, inadequate access to personal protective equipment and moral distress.^18^ Our survey found working in front-line settings was not associated with increased risk of psychological distress. This could be because distress related to COVID-19 extended beyond one’s occupational exposure risk, which at the time of the survey was low, to a general preoccupation with the pandemic, its uncertain future course, socio-economic and lifestyle impacts, exposure to media and the limitation of social supports.^5^ This highlights the importance of making supports flexible and available to all HCWs, not just front-line workers.

Data from previous epidemics and abroad during COVID-19 have identified disparate mental health impacts amongst different professionals.^5,9^ In our study, senior medical staff reported lower rates of psychological distress than other staff. This is consistent with *beyondblue* survey data, showing senior doctors report less psychological distress than their less senior colleagues.^2^

There is limited research examining the relationship between mental health outcomes and a pre-existing mental illness during the COVID-19 pandemic. Cardozo et al.^19^ reported that a history of mental illness increased the risk of psychological distress following deployment in humanitarian workers. Our study supported this finding, identifying past psychiatric history as predictive for reporting symptoms of anxiety, depression, PTSD and burnout.

This study also investigated the relationship between resilience, burnout and symptoms of psychological distress. The relationship between burnout and mental illness remains unclear;^4^ however, recent research suggests burnout increases the risk of developing depression and PTSD.^6^ In our study, 29.5% of participants screened positively for burnout, and symptoms of burnout were predictive of psychological distress.

Psychological resilience mediates the stress response to trauma. High levels of psychological resilience are protective against the development of mental illness,^20^ and this was reflected in this study. Recent research has considered introducing resilience training as a preventative treatment for reducing mental health outcomes amongst first responders.^20^ Similar strategies could be developed for HCWs in anticipation of future public health emergencies.

Our study had some limitations. Cross-sectional studies don’t allow tracking of changes in psychological distress following the onset and escalation of the pandemic. Our dissemination strategy precluded a formal response rate calculation. A large number of responses were excluded due to incomplete data. Selection bias and response bias may have resulted in an overestimation or underestimation of psychological distress and rates of pre-existing psychiatric history.

The study highlights the importance of mental health support during and following the COVID-19 pandemic. Future research should consider long-term mental health outcomes and burnout in front- and second-line workers, and in junior and senior clinicians. It should examine the factors underlying these with the aim of developing effective interventions.
